# Randomized Trial of a Pharmacist-Delivered Intervention for Improving Lipid-Lowering Medication Adherence among Patients with Coronary Heart Disease

**DOI:** 10.1155/2010/383281

**Published:** 2010-08-17

**Authors:** Yunsheng Ma, Ira S. Ockene, Milagros C. Rosal, Philip A. Merriam, Judith K. Ockene, Pritesh J. Gandhi

**Affiliations:** ^1^Division of Preventive and Behavioral Medicine, Department of Medicine, University of Massachusetts Medical School, 55 Lake Avenue North, Worcester, MA 01655, USA; ^2^Division of Cardiovascular Medicine, Department of Medicine, University of Massachusetts Medical School, Worcester, MA 01655, USA; ^3^Global Medical Affairs, Genzyme, Cambridge, MA 02142, USA

## Abstract

A randomized trial of a pharmacist-delivered intervention (PI) versus usual care (UC) was conducted; 689 subjects with known coronary heart disease were recruited from cardiac catheterization laboratories. Participants in the PI condition received 5 pharmacist-delivered telephone counseling calls post-hospital discharge. At one year, 65% in the PI condition and 60% in the UC condition achieved an LDL-C level <100 mg/dL (*P* = .29); mean statin adherence was 0.88 in the PI, and 0.90 in the UC (*P* = .51). The highest percentage of those who reached the LDL-C goal were participants who used statins as opposed to those who did not use statins (67% versus 58%, *P* = .05). However, only 53% and 56% of the patients in the UC and PI conditions, respectively, were using statins. We conclude that a pharmacist-delivered intervention aimed only at improving patient adherence is unlikely to positively affect outcomes. Efforts must be oriented towards influencing physicians to increase statin prescription rates.

## 1. Introduction

Cardiovascular disease (CVD) is a leading cause of mortality worldwide. An estimated 79 million Americans have CVD, of whom almost 16 million (20%) have coronary heart disease (CHD) [1]. Secondary prevention efforts aimed at treating risk factors such as dyslipidemia have reduced the morbidity and mortality related to CVD [2]. Numerous studies have demonstrated a reduction in mortality with the use of 3-hydroxy-3-methylglutaryl-coenzyme A reductase inhibitor (statin) therapy [3–7]. The serum low-density lipoprotein cholesterol (LDL-C) goal, as reflected in the treatment guidelines of the Adult Treatment Panel of the National Cholesterol Education Program (NCEP ATP-III) [8], is <100 mg/dL. An update of the guidelines in 2004 supports an LDL-C goal of 70 mg/dl for secondary prevention [4]. 

Critical to the success of any medication is adherence to the prescribed dosage regimen. However, long-term adherence for many medications is poor. Prior studies of preventive interventions have documented inadequate adherence in approximately 50 percent of patients [9–11]. Significant numbers of patients either never fill or do not take their medications as prescribed. Among those who do initially take the prescribed medications, many patients discontinue medications over time. Observed discontinuation rates are directly proportional to length of time on the medication, and range from 15% to 74% [12–16]. None of these studies include patients who are of status post a coronary event or procedure. 

While statin medication use has increased in the last decade, use and adherence to statins remains suboptimal [12, 17, 18]. Using the National Health and Nutrition Examination Survey (NHANES) 2003-2004, Mann and colleagues reported statin use by only 19.6% of US adults with high LDL-C levels in 1999-2000, 27.3% in 2001-2002, and 35.9% in 2003-2004, suggesting increasing but still suboptimal use (*P*-value for trend <.001) [17]. In a study of New Jersey Medicaid data, only 9.2% of patients filled a statin prescription within 90 days of hospital discharge for acute MI in 1995 as compared to 43.1% of patients in 2003 (*P*-value for trend <.001), however, only 25% of those who filled an initial prescription continue to take statins 5 years after initiation of therapy [12]. In a more recent analysis from Canada, 2-year adherence rates ranged from 48% for fluvastatin to 63% for atorvastatin [18]. 

Pharmacist-delivered interventions may improve adherence to prescribed lipid-lowering medications. The Pharmacist Assisted Compliance Trial (PACT, ClinicalTrials.gov Identifier: NCT00848224) was a randomized controlled trial testing a pharmacist-delivered program to improve adherence to lipid-lowering pharmacologic therapy in patients with known CHD [19]. The objective of this study was to compare intervention and usual care conditions for LDL-C goal attainment and proportion of prescribed lipid-lowering medication taken by subjects over a one-year period.

## 2. Materials and Methods

### 2.1. Study Design and Population

The overall goal of this study was to implement and evaluate the effects of a pharmacist-delivered intervention (PI) designed to improve LDL-C goal attainment according to the NCEP ATP-III Guidelines and prescribed lipid-lowering medication adherence in patients with known CHD. In this two-condition randomized clinical trial, the intervention condition included: (1) a computer-based tracking system designed to facilitate follow-up of patients who were initially seen for a CHD clinical event at UMass Memorial Medical Center (UMMMC); (2) an initial inpatient contact and a series of coordinated patient-centered pharmacist-delivered telephone counseling contacts to improve adherence to prescribed medications. The tracking system was developed based on a communication system using Lotus Notes from IBM (Lotus Notes R5.0.11). Eligibility data, demographic information, and contact and proxy contact information were entered into the system. The system was shared by the study team. We used it to track eligible and ineligible patients, and facilitate timely scheduling of assessments and intervention sessions. Multiple levels of password protection were utilized to ensure data security. The pharmacists also provided feedback and recommendations to the patients' physicians and nurse practitioners. The pharmacist had access to the lipid values during the study.

The study population consisted of 689 patients recruited from the cardiac catheterization laboratories of UMMMC, a tertiary care hospital in central Massachusetts. A patient was eligible for the study if he/she was between the ages of 30 and 85 years and had CHD defined as the presence of at least one coronary lesion ≥50% at the time of coronary angiography. Patients could have a history of prior CHD, or this could have been their first such diagnosis. Patients were excluded if they were unable or unwilling to give informed consent in English, had a history of intolerance to two or more statin drugs, planned to move out of the area within one year of recruitment, had a poor prognosis such that life expectancy was estimated to be <5 years, had a major psychiatric illness, or had no telephone. 

Patients were randomly assigned to the UC condition or to the PI condition. The UC condition consisted of normal clinical care as determined by the patient's provider. Patients in the PI condition were seen by one of the study pharmacists prior to discharge. This allowed the pharmacist to establish a relationship with the patient, explain the pharmacist's role in the study, provide education about all discharge medications including a medication card which listed all medications and their manner of use, and set the framework for the follow-up telephone calls. The pharmacist-delivered telephone counseling calls took place at two weeks, and at 1, 3, 6, and 9 months following discharge. The goal was to assist patients to remain adherent to prescribed statins and other medications, and also promote adherence to AHA guidelines for LDL-C. During these calls, pharmacists utilized a patient-centered counseling algorithm (address general issues, assess, advise, assist, arrange follow-up; see the appendices for details) to help patients develop a medication adherence plan. In addition, the pharmacist facilitated scheduling of repeat blood draws for lipid measurement and provided information, guidelines; and prompts to the patient and to the patient's physician or nurse practitioner with regard to LDL-C management. Specifically, the provider received an emailed summary of the discussion after each pharmacist contact with the patient. The email included three categories (i.e., adherence, CAD, and hyperlipidemia) along with recommendations for each. At the in-patient visit, patients in the PI condition were also provided an educational packet, a dietary goal booklet, and a pillbox. Participants in the PI condition also were mailed updated medication cards if their medication regime had changed. 

The study pharmacists were trained in the delivery of patient-centered counseling [20, 21] and followed patient-centered protocols for the in-patient and telephone contacts. This training included a 4-hour meeting that presented pharmacists with an orientation to the study (i.e., background, design, and timeline), the theoretical framework for the intervention, patient-centered counseling protocols and opportunities for role-playing the protocols among themselves. An additional one-hour role-playing session was completed within 2 weeks of the first training session, and provided further opportunities for role-playing potential patient scenarios. During the individual session the pharmacists were provided with immediate feedback regarding their counseling skills, and had an opportunity to correct problems. For quality control, one of the investigators (Dr. Milagros C. Rosal) listened to telephone call recordings monthly for the first 2 months and then bimonthly through year 2. Two booster sessions were conducted within the 2 years of the study intervention, providing the pharmacists with feedback on their counseling skills and eliciting discussion of challenges encountered with patients. The pharmacist was told of the quality control procedures and verbal informed consent was obtained from the patient when a call was recorded.

The patient was the unit of randomization and analysis. Randomization was conducted by a statistician who was not involved with the intervention. The study was conducted between September 2000 and August 2005. The Institutional Review Boards of the University of Massachusetts Medical School approved all subject recruitment, intervention, and data collection procedures.

### 2.2. Data Collected

Data collected included a serum LDL-C level at 12 months and pharmacy refill information between the baseline and one-year study participation dates. Pharmacy refill records were obtained from all pharmacies where each patient obtained their medications. They were our primary measure for ascertaining patient adherence to statin, angiotensin-converting enzyme (ACE) inhibitor, and beta-blocker medications. The number of diseased vessels as measured by coronary angiography was obtained from each patient's catheterization report. Patient demographic characteristics were measured at baseline. Data measured at one year included a blood lipid profile, a 24-hour diet and physical activity recall, smoking status, and measures of height, weight, and waist circumference. 

### 2.3. Outcomes

*The primary outcome* evaluated at one year included percentage of patients with a serum low-density lipoprotein cholesterol (LDL-C) level <100 mg/dl; *the secondary outcome* included the proportion of prescribed statin medication taken by patients as measured by a continuous multiple-interval (CMA) based on pharmacy records. The CMA is the ratio of days supply obtained to total days between refill records [22]. Other secondary outcomes evaluated at one year included the proportion of patients prescribed ACE inhibitor and beta-blocker medication. Adherence to these medications was also measured by CMA. 

### 2.4. Sample Size

The sample size chosen was a balance between cost/resources, study complexity, and statistical testing power. With 345 patients in each condition, this study had sufficient power (90% or greater) to detect a change of 10 percentage points in the rate of subjects achieving the LDL-C goal of <100 mg/dl between two conditions even under the most conservative conditions. This sample size also allowed sufficient power to detect a change in mean CMA of 0.12 even in a conservative scenario of 40% on therapy in each condition.

### 2.5. Statistical Analysis

The dichotomous value of achieving the goal LDL-C was compared between the two randomized conditions using a Fisher's exact test with an alpha level of 0.05 used for statistical significance. Since the distribution of CMA values was approximately normal, it was compared using a *t*-test between two conditions. Logistic regression analysis was conducted for predicting LDL-C goal by intervention group adjusted for covariates such as age, gender, and statin medication adherence.

## 3. Results

**Figure 1:**
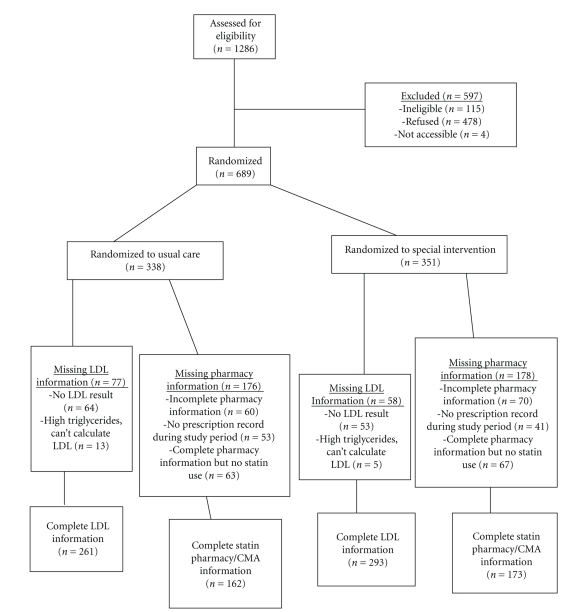
Study recruitment, follow-up, and sample used for the PACT analysis.

Of 689 patients recruited, 338 were randomized to the control condition and 351 to the intervention condition. A total of 559 (81%) had complete pharmacy records and were included in the final analysis. There was no statistically significant difference between intervention and control subjects in the percentage of subjects with complete pharmacy records (80% versus 82%, *P* = .50). Of the total patient population, 554/689 or 80.4% had a one-year LDL-C value available; 83.5% in intervention versus 77.2% in the control condition. This difference was due to a greater number of subjects in the control condition having triglycerides ≥400 mg/dl, precluding the calculation of LDL-C. In total, 65.5% of the participants had both pharmacy and LDL-C measures, with no significant difference between the conditions (66.4% versus 64.5% for intervention versus control, resp.).  Figure 1 is a flowchart that outlines the recruitment, follow-up, and sample used for the analysis.

The mean age of participants was 60 years, with an average body mass index (BMI) of 30 kg/m^2^. Sixty percent were male, 68% were married or lived with partner, and 90% were white. Forty-five percent had a high school or less education (Table 1). Participants in the UC condition were more likely to be smokers, but were not statistically different from the PI participants in any other characteristic, including demographics, BMI, and catheterization-assessed severity index [23, 24]. 

Table 2 presents the results of lipid measures. At 12 months, 65% achieved the goal LDL-C level of <100 mg/dl in the PI condition, and 60% achieved this in the UC condition (*P* = .29). Average levels of total cholesterol, HDL-C, LDL-C, and triglycerides also were similar between the two conditions. 

Three hundred and thirty-two subjects (48%) used statin therapy during the study. Of these 85% used atorvastatin, 13% simvastatin, 5% pravastatin, 2% fluvastatin, and 1% lovastatin. Seventy-four percent of statin refills were written for a 30-day supply. At one-year, only 53% and 56% of the participants in the UC and PI conditions, respectively, were using statin medication. Use of nonstatin lipid-lowering medication was infrequent, and these medications included fenofibrate (9 patients), gemfibrozil (7 patients), slow-release niacin (6 patients), colesevelam (4 patients), and cholestyramine (1 patient). 

Table 3 presents adherence with statin medications. The CMA for statin medication use was 0.88 (standard deviation (SD) = 0.3) in the PI condition (referring to the patient being 88% adherent to their statins medication), and 0.90 (SD = 0.3) in the UC condition (*P* = .51). The number of statin refills and total days on statins were similar in the two conditions. 

Adherence to beta-blockers and ACE inhibitors is also noted in Table 3, showing no statistical differences in the use of these medications in the two conditions. The number of beta- blocker and ACE inhibitor refills was also similar in the two conditions, and, as was the case with statin therapy, very high (>90%) in all conditions.

Multivariate logistic regression model results predicting LDL-C <100 mg/dl included variables of study condition, gender, age, and CMA. Results indicated that females were less likely to reach the LDL-C goal (odd ratio = 0.62, 95% confidence interval: 0.39–0.97, *P* = .04); and patients aged 61–70 were more likely to reach goal (odd ratio = 2.27, 95% confidence interval: 1.18–4.34, *P* = .01, referent group: age < 51). Patients with greater statin CMA levels also had a higher likelihood of attaining the LDL-C goal (odd ratio for CMA > 1 = 2.23, 95% confidence interval: 1.17–4.24, *P* = .01, referent group: CMA = 0). Study condition was not significantly associated with LDL-C goal attainment. We considered several other variables such as depression, however, they were not significantly predicting LDL goal, thus were not included in the final model.

Since a smaller proportion of females reached LDL goals, we examine differences in the measurements by gender. Compared to men, women were on average older, had higher BMI, had lower education level, were less likely to be married/live with a partner, and had a lower coronary disease severity score. Women were more likely to use a pillbox, took medication more often, and were more likely to believe that spirituality influences health. 

We examined statin medication use and LDL-C outcome. The highest percentage of those who reached the LDL-C goal of <100 mg/dl were patients who used statins as opposed to subjects who did not use statins medications (67% versus 58%, *P* = .05). They also had lower LDL-C levels at one-year (93 mg/dl for statins use versus 99 mg/dl for non-statins use, *P* = .04). 

Completion rates for pharmacist counseling calls were high: 78% for 2 week calls; 80% for 1 month calls; 78% for 3 month calls; 72% for 6 month calls; 70% for 9 month calls. Forty-eight percent completed all 5 calls and 62% completed at least 4 calls. There was no apparent relationship between intervention dose (i.e., number of intervention calls, the total minutes of call time, and the average minutes of call time) and the study outcomes (i.e., adherence or LDL-C level).

## 4. Discussion

Approximately two-thirds of subjects reached their LDL-C goal one year after enrollment. There was no significant difference between the PI and UC conditions in LDL-C goal attainment or statin medication adherence. However, the adherence rate of the control condition was unexpectedly high, and the level of adherence attained (CMA of 90%) made further improvement by the intervention condition difficult to attain. Equally high adherence rates were seen for beta-blocker and ACE inhibitor medications. These high adherence rates may reflect a high level of patient concern following catheterization for CAD, or increasing awareness of and attention to medication adherence on the part of medical staff.

Compared to other reports on adherence to medications that show a small percentage of subjects reaching their LDL-C goal, a greater percent of our study participants (over 60%) both reached their LDL-C goal and adhered to statin prescriptions. However, failure to reach LDL-C target levels remains common. Sueta and colleagues analyzed data from a retrospective chart audit of 48,586 adult outpatients with CHD from 140 medical practices (80% cardiology only) [25]. Of these patients, only 44% had annual diagnostic testing of LDL-C, and of this 44%, only 25% (11% of the total) achieved the target LDL-C level of <100 mg/dl. Only 39% of patients were taking lipid-lowering therapy, and appropriate adherence was less common among the older than among the younger patients. 

Cohen and colleagues followed up on patients who had been hospitalized for chest pain and found to have CHD [26]. At one-month, only 17% of those with high levels of total cholesterol or LDL-C were being actively treated with either diet or drugs. At one to two year follow-up that percentage had reached 35%. The percentage who actually reached the goal LDL-C level was unknown. In a study of 825 men and women with CHD followed at 16 academic medical centers, at the end of a three-year period of follow-up 55% of the men but only 35% of the women were on pharmacologic lipid-lowering therapy. The target LDL-C goal of less than 100 mg/dl was achieved in only 31% of the men and only 12% of the women. The authors suggest that a gender bias may exist in the use of such therapy [27]. According to the recent NHANES, statins were being used by only 19.6% of US adults with high LDL-C levels in 1999-2000, it was increased to 27.3% in 2001-2002, and 35.9% in 2003-2004, respectively (*P* value for trend <.001). LDL-C goal was achieved by 49.7%, 67.4%, and 77.6% of statin users in 1999-2000, 2001-2002, and 2003-2004, respectively (*P* trend <.001) [17]. Thus the large majority of patients with known CHD or CHD risk are inadequately treated for hyperlipidemia or, if treated, do not adhere to the regimen. Reasonable estimates based on the literature would suggest that no more than 30–40 percent are treated and if treated, many do not adhere to lipid-lowering medication, such that somewhere between only 15 and 30 percent achieve an LDL-C goal level of <100 mg/dl [17, 25–27]. 

Other trials have demonstrated the effect of pharmacist intervention on medication adherence. Erickson and colleagues found that over an average five-month follow-up, significant decreases in mean blood pressure were noted for patients who were monitored by a clinical pharmacist and a physician as compared to patients who received care from a physician alone [28]. In this study, the pharmacist made recommendations regarding pharmacotherapy to physicians whose patients were in the intervention condition, while in the control condition, physicians received no such education, and interventions relating to pharmacotherapy were only physician initiated. In a similar study conducted recently, Borenstein and colleagues compared the effectiveness of an evidence-based systematic approach to hypertension care involving comanagement of patients by primary care physicians and clinical pharmacists versus usual care in reducing blood pressure in patients with uncontrolled hypertension. The study also resulted in improved blood pressure control and reduced average visit costs/patient [29]. 

In a crossover study design, Lee and colleagues also found that a pharmacy care program led to increases in medication adherence, medication persistence, and clinically meaningful reductions in blood pressure, whereas discontinuation of the program was associated with decreased medication adherence and persistence [30]. 

Although adherence with statin medications was higher than expected [22], physician prescribed statins prescriptions and dosages were often inadequate. Approximately 40% of patients did not achieve an LDL-C level of <100 mg/dl in this study. In addition, only 53% and 56% of the patients in the UC and PI conditions, respectively, were prescribed statin medication. The relatively low percentage of patients prescribed statin medication may explain why many participants did not reach their LDL-C goal. 

This study has several strengths. It is a randomized controlled clinical trial that collected detailed information on medications and demographics. CMA was used to calculate medication adherence. It accounts for discontinuation and gaps between refills. Other methods of assessing adherence are available, but tend to overestimate medication consumption (pill counts) or are expensive and intrusive (electronic monitors) [22]. 

There are several limitations of the study. First, although the sample size of 689 in this study was sufficient to provide ample power to detect outcomes, the sample size available for LDL-C outcome was smaller (293 for the PI control and 260 for the control condition), and the power to detect what level of LDL-C difference was decreased to 70%. This is an important limitation. However, this study is one of the largest clinical trials on this topic ever conducted. Second, we used pharmacy refill data that described the dispensing of medication, but had no other available information to indicate whether dispensed medications were actually taken by the patients. Likewise, we have no data on patients who may have been prescribed medications that they never filled. Third, we did not have data on cost of the medication and insurance coverage, which also affects medication adherence. Fourth, our intervention focused on patients' medication adherence; although pharmacists made recommendations about putting patients on statins to patients' primary care physicians via telephone, e-mail, and letters, this part of intervention was not a primary focus of the study. Therefore, no data are available as to whether physicians followed the pharmacists' recommendations. However, two previous studies demonstrated the effect of pharmacist intervention on hypertensive medication adherence, in which pharmacists made recommendations to physicians regarding pharmacotherapy for patients with uncontrolled hypertension. Both trials concluded that hypertension care involving co-management of patients by primary care physicians and clinical pharmacists resulted in improved blood pressure control and medication adherence [28, 29]. In light of the lack of differences seen with the primary study endpoints in the current study, future trials should consider evaluating interventions that link pharmacists to physicians in an attempt to influence physicians' patterns for prescribing lipid-lowering pharmacotherapy. Patients' cardiologists were not contacted in this study. This could be an area of further research, i.e., would contact with the cardiologist have led to a greater intervention effect? Fifth, lipid levels fall rapidly after an acute CHD event [31] and we did not have LDL-C values at baseline. We do not know how many patients met criteria for prescription for statin medication based on the guideline at the time of their enrollment in the study. Sixth, the majority of patients in this study were Caucasian, thus limiting our ability to generalize to other populations. Finally, one possibility for the high adherence rate seen in the control condition may be due to selection bias. Unfortunately, we do not have complete demographic information to be able to compare participants and nonparticipants. It is possible that there are selection biases that we are not able to describe. By the very nature of a randomized controlled trial, requiring a consent process and either formally or informally excluding individuals who have memory impairment or who are unable for a variety of reasons to provide consent postcatheterization, a selection bias exist that favors the inclusion of individuals more likely to be adherent to pharmacologic therapy.

## 5. Conclusions

A pharmacist-delivered intervention aimed only at improving patient adherence to prescribed lipid-lowering medications was unsuccessful in attaining target AHA-recommended LDL-C goals. Achieving the goal of increasing adherence to lipid-lowering pharmacotherapy was precluded by very high adherence rates in the control condition. However, statin prescription rates were lower than desirable. Future pharmacist-delivered trials may be more effective if they are oriented towards influencing physicians' prescribing patterns, regarding prescription of appropriate medication. Further studies of medication adherence also need to concentrate on populations more likely to have difficulties with medication-taking behavior. 

## Figures and Tables

**Table 1:** Demographics of participants, Pharmacist Assisted Compliance Trial, Worcester Massachusetts, 2000–2005 (*n* = 689).

Condition	Control		Intervention		*P*-value
Continuous Variable					

	Mean (SD)	*n*	Mean (SD)	*n*	
Age	60.29 (10.4)	338	60.38 (10.5)	351	.905
BMI from Catheterization Report	30.05 (5.7)	318	30.33 (5.8)	328	.536
BMI self report 1 year	30.11 (5.3)	239	29.81 (5.7)	253	.542
Change in BMI	−0.03 (2.9)	226	−0.42 (3.0)	239	.149
Catheterization Report Severity Index^a^	1.78 (0.9)	321	1.88 (0.9)	336	.189

Categorical Variable					

	% (Frequency)		% (Frequency)		
Gender: Female	40.24 (136)	338	40.17 (141)	351	1
Smoking status: Smoked within 1 year	27.76 (78)	281	20.40 (61)	299	.041
Education: High School or less	45.91 (118)	257	45.20 (127)	281	.931
Race: white	90.53 (306)	338	88.89 (312)	351	.532
Marital status: Married or living with partner	67.78 (162)	239	67.70 (174)	257	1
Employment: Fulltime or part-time	52.61 (151)	287	52.90 (164)	310	1

^a^Cath Report Severity Index was calculated based on the number of diseased arteries [23, 24].

**Table 2:** Cholesterol measure at one-year, Pharmacist Assisted Compliance Trial, Worcester Massachusetts, 2000–2005 (*n* = 689).

Condition	Control (*n* = 338)		Intervention (*n* = 351)		
	% (Frequency)	*n*	% (Frequency)	*n*	*P*-value
LDL-C <100 goal	60.15 (157)	261	64.51 (189)	293	.293
LDL-C <70 goal	18.77 (49)	261	17.06 (50)	293	.657

	Mean (SD)		Mean (SD)		
Total Cholesterol	172.85 (48.6)	274	167.27 (39.3)	297	.13
1 year LDL-C	97.79 (35.1)	261	94.47 (31.3)	293	.24
1 year HDL-C	42.61 (11.4)	272	42.30 (12.3)	297	.755
1 year Triglyceride	137.00 (8.96)	270	130.32 (8.87)	296	.29

**Table 3:** Statins, Beta-Blockers, and ACE Inhibitor medication compliance at one-year, Pharmacist Assisted Compliance Trial, Worcester Massachusetts, 2000–2005 (*n* = 689).

Condition	Control (*n* = 338)		intervention (*n* = 351)		*P*-value
	Mean (SD)	*n*	Mean (SD)	*n*	
*Statins*					
Number statin refills	6.13 (3.7)	179	6.35 (3.8)	196	.562
Statin CMA	0.90 (0.3)	162	0.88 (0.3)	173	.509
Number of prescriptions	2.41 (0.8)	223	2.55 (0.8)	238	.078
Total days on statin	290.53 (94.30)	179	291.20 (92.40)	196	.944

*Beta Blockers*					
Number refills	5.80 (4.0)	189	6.34 (4.0)	207	.179
Beta-blocker CMA	0.91 (0.4)	157	0.97 (1.1)	180	.557
Total days on beta-blocker	283.19 (101.50)	189	287.55 (99.40)	207	.667

ACE Inhibitors					
Number refills	5.73 (3.8)	146	6.23 (3.9)	179	.247
ACE CMA	0.93 (0.3)	126	1.02 (0.8)	157	.249
Total days on ACE	280.94 (93.40)	146	283.56 (95.00)	179	.803

	% (Frequency)		% (Frequency)		
Monthly^a^ statin prescription	69.83 (125)	179	70.41 (138)	196	.911
Monthly^a^ beta-blocker prescription	59.79 (113)	189	64.25 (33)	207	.407
Monthly^a^ ACE prescription	61.64 (90)	146	70.39 (126)	179	.1

∗a: An indicator of whether all prescriptions for the medication were monthly (or less) prescriptions (versus 90 day or something >30 days at any time).

## Appendices

### A. Patient-Centered Counseling Algorithm: In-Patient Visit

*Medication adherence should be assessed and emphasized for statins (HMG-COA reductase inhibitors), β* *-blockers, and ACE-inhibitors*

Introduction Hello, I am Dr. __________. I am the pharmacist at the hospital.

Assess Is this a good time to talk about your medicines? I understand that you have been told that you have heart disease.

(1) Knowledge

What do you know about the role of cholesterol in heart disease? What do you know about the role of diet and medication in cholesterol control?

(2) Past experiences

Have you taken cholesterol-lowering medication in the past? What medications do you take at home? What type of difficulties have you encountered with regard to taking your medications in the past? How did you deal with them? Many people have difficulties with __________ (go through the “problems” checklist). Have you experienced any of these?

(3) Motivations/Concerns

How do you feel about taking __________? It is helpful to identify your own reasons for doing something. What are some reasons that you might have for taking the medication? What concerns you about taking __________?

Advise

(1) Encouragement

I strongly advise that it is important for your health that you take __________ each day as __________ (state medication regimen.)

(2) Tailored advice

The benefits of taking __________ in your case can prevent you from experiencing a heart attack.

Assist

(1) Correct misunderstandings

Some of the possible side effects of this medication are __________. Based on our discussion so far, what questions do you have?

(2) Provide support

Provide resources and materials (information packet on diet, medication information, medication sheet and pillbox). I know it is sometimes hard to take (additional) medications. I'd like to help you with this. I'm optimistic that you'll be able to take your medication and reduce your cholesterol level.

(3) Behavioral strategies

It is important that you take __________ at these times __________ every day (state the goal for each medication).

(4) Stimulus control

What types of cues could you use to remind yourself to take your medication? Based on past experiences, are there any other situations that could make it difficult for you to take your medication in the future? Keeping track of how often you take your medication is very important. What might help you keep track of whether or not you've taken your medication each day?

(5) Address Problems/concerns

You mentioned that __________ (state potential challenges to medication intake identified by the patient) could potentially interfere with your taking your medication. Can you think of ways to deal with each of these problems? What could you do to prevent this situation form affecting your ability to take your medication? (State one potential problem at a time). Consider a written behavioral contract. Encourage use of support (medication sheet, pillbox, other people). What or who might help you? How will this be helpful to you? So that I know that I have not forgotten to tell you everything about your medicines, please tell me how you are going to take __________? As we discussed, some things that help you take the medication as intended are __________ (mention discussed strategies to enhance compliance). I just want to take a minute to review the behavioral contract with you. What medication-related questions do you have for me?

Arrange Follow-up I would like to call you to see how you are doing. What is the best time (day of week and time of day) to call you? Schedule telephone calls and provide the patient with the list of dates and times the pharmacist will be calling (2 weeks, 4 weeks, 12 weeks, 24 weeks, and 36 weeks). Ask the patient to have all their medications and behavioral contract before them prior to the pharmacist call.

### B. Patient-Centered Counseling Algorithm First Telephone Call (2 weeks)

*Medication adherence should be assessed and emphasized for statins (HMG-COA reductase inhibitors), β* *-blockers, and ACE-inhibitors.*

Introduction Hello, this is Dr. __________ calling from the UMass Memorial Medical Center. Can I please talk to Mr./Ms. __________ (state name of patient)? Mr./Ms. __________, we met about two weeks ago while you were in the hospital.

Assess

(1) General issues

How have you been doing? Is this a good time to talk about your medications? Do you have any questions about the role of cholesterol in heart disease? Do you have all your medications and the behavioral contract in front of you?

(2) Recent Experiences with Medications

How are you doing with your medications? How do you take __________? What questions do you have about your medications? The last time we talked, you mentioned that __________ (name the problem the patient identified in the in-patient visit) had the potential to interfere with your taking your medications. Was __________ a problem for you in the past 2 weeks? How did you deal with this (ask this question for each problem reported by the patient)? Have you experienced any other difficulty with regard to taking your medications?

(3) Motivations/Concerns

How do you feel about taking __________? (Do this for statins, beta-blockers and ACE inhibitors). It is helpful to identify your own reasons for doing something. What are some reasons that you might have for taking the medication? Do you have any concerns about taking __________? (Do this for statins, beta-blcokers, and ACE inhibitors).

Advise

(1) Encouragement

I strongly advise that it is important for your health that you take __________ each day as prescribed by your health care provider.

(2) Tailored advise

The benefits of taking __________ in your case can prevent you from experiencing a heart attack.

Assist

(1) Provide support

I know it is sometimes hard to take (additional) medications. I'd like to help you with this. I'm optimistic that you'll be able to take your medication and reduce your cholesterol level.

(2) Address problems/concerns

You mentioned that __________ (state any new challenges to medication intake identified by the patient during this call) interferes with your taking your medication. Can you think of ways to deal with __________ (state barrier)?

(3) Behavioral strategies

Review behavioral contact (what medications, when and how taken, potential challenges, and ways to deal with them, cues to remind patient of medications, tracking medications intake). Is there anything else that can be helpful to you? What medication-related questions do you have for me?

Arrange Follow-up According to my schedule, I will call you __________ (specify date and time of day) to see how you are doing. Is this a good time? Schedule next telephone call. Remind patient to have their medications and behavioral contract in front of them before the next telephone call.

### C. Patient-Centered Counseling Algorithm: Follow-up Telephone Calls

*Medication adherence should be assessed and emphasized for statins (HMG-COA reductase inhibitors), β* *-blockers and ACE-inhibitors. Refill records and fasting lipid profiles should be obtained prior to the 12-week, 24-week, and 36-week calls. If necessary, please mail a revised medication sheet and behavioral contract to the patient after each follow-up telephone call.*

Introduction Hello, this is Dr. __________ calling from the UMass Memorial Medical Center. Can I please talk to Mr./Ms. __________ (state name of patient)?

Assess How have you been doing? Is this a good time to talk about your medications? Do you have all your medications and the behavioral contract in front of you? The last time we talked, you were taking __________. Are you still on this medication? What do you take it for? How do you take it? What problems have you had with taking this medication? Do you have any concerns about taking this medication? How do you handle __________ (state problem/concern). *Based on refill records, if the patient has switched from one agent to another, or if the dose has been changed then acknowledge the change and ask the questions above. *

Advise Given your high cholesterol (and other risk factors), I strongly advise you to continue taking your medications as __________ (state drug regiment). The benefits of taking __________ in your case can prevent you from experiencing a heart attack.

Assist The last time we talked you said that __________ (state challenges/potential barriers) was a problem? How did you deal with this? What could you do in the future if/when you lapse in medication taking? Keeping track of or monitoring how often you take your medications is important. How are you keeping track of the medications that you take? *Refer to behavioral contract. Encourage patient to use pillbox and medication sheet. *
So that I have not forgotten to tell you everything, please tell me how you are going to take __________? As we discussed, some things that help you take the medication as intended are __________ (mention strategies to enhance compliance). What medication-related questions do you have for me?

Arrange Follow-up According to my schedule, I will call you __________ (specify date and time of day) to see how you are doing. Is this a good time? Schedule next telephone call. Remind patient to have their medications and behavioral contract in front of them before the next telephone call.
